# Focused molding using adhesive pads in Mehta casting for early-onset scoliosis

**DOI:** 10.1186/1748-7161-9-S1-O36

**Published:** 2014-12-04

**Authors:** Roby Abraham, Paul Sponseller

**Affiliations:** 1 SUNY Downstate Medical Center, Brooklyn, NY, USA; 2 The Johns Hopkins University, Baltimore, MD, USA

## Background

Early-onset scoliosis is often effectively managed by serial casting. Properly localizing the apex of the molds with the cast in place is challenging. The authors explored the effectiveness of a novel technique: incorporation of adhesive pads placed over the major curve apex before Mehta casting.

## Aim

To determine the effect of adhesive pads placed over the apex of scoliosis curves on curve correction 1) after the first cast and 2) after final cast.

## Study design

Case series.

## Method

The 27 patients who received body casts (2000 through 2013) were divided into 2 groups, those without and with apical adhesive pads (5 to 6 layers of pads placed on the major curve’s apex during casting): non-pad (NP) group (n = 12) and pad (P) group (n = 15), respectively. Groups were compared for percentage of Cobb angle change from the first cast and curve correction to a Cobb angle of less than 25° with Student t and chi-square tests (significance, p value less than .05).

## Results

The mean percentage of major first-cast curve correction was 39% ± 18% and 56% ± 17% in the NP and P groups, respectively. Of the 26 patients out of cast, 11 (42%) had a Cobb angle of less than 25°: 3 (25%) and 8 (57%) in the NP and P groups, respectively. The mean differences between the 2 groups in percentage of major curve correction and this Cobb angle correction were significant: p = 0.023 and 0.005, respectively.

## Conclusion

Adhesive pads placed over major curve(s) during Mehta casting were effective in increasing the amount of major curve correction from the first cast for idiopathic early-onset scoliosis, and in decreasing curves to less than 25° at final follow up.

## Level of evidence

Level III; Retrospective comparative study.

